# Identifying high-risk groups for self-harm in adolescents using the Avon Longitudinal Study of Parents and Children (ALSPAC): a cross-cohort comparison latent class analysis study

**DOI:** 10.1007/s00787-025-02702-z

**Published:** 2025-04-05

**Authors:** David McEvoy, Ross Brannigan, Colm Healy, David Mongan, Mary Clarke

**Affiliations:** 1 School of Population Health, Royal College of Surgeons Ireland (RCSI), Beaux Lane House, Mercer Street Lower, Dublin 2, Ireland; 2 Division of Psychiatry, Centre for Clinical Brain Sciences, University of Edinburgh, Edinburgh, UK; 3 Centre for Public Health, Queen’s University Belfast, Belfast, Northern Ireland UK; 4 Department of Psychiatry, Royal College of Surgeons in Ireland, Dublin, Ireland

**Keywords:** Self-harm, Self-injury, Sub-groups, Adolescents, Young adults, ALSPAC

## Abstract

Young people who self-harm are at an increased risk of suicide. Furthering our understanding of the risk factors for self-harm is essential for identifying high-risk groups, which can be used to inform the design of preventative interventions. This study used the Avon Longitudinal Study of Parents and Children (ALSPAC) and applied latent class analysis to the risk factors for self-harm at ages 13 and 17. Longitudinal associations between the latent classes and self-harm at ages 17 and 20 were examined. Cross-cohort comparisons were conducted between this study and a previous study using Irish data. At age 13 there was a low risk group, a peer problems group, and substance use group, similar for the two cohort studies, and a family conflict group, which was the least similar group to its matching group in the Irish study. All of these age 13 high-risk groups had approximately twice the relative risk (between 1.3 and 2.5) for self-harm at age 17 compared to the low risk group. The age 17 models were very similar across the two cohorts, each with a low risk group, a depression and high substance use group, a depression and low substance use group, and a substance use group. The relative risk of self-harm at age 20 for these high-risk groups compared the low risk group ranged from 3.6 to 8.0. These groups could help identify those at risk of self-harm and inform the design of prevention programmes to reduce self-harm behaviour in young people.

**Supplementary Information:**

The online version contains supplementary material available at 10.1007/s00787-025-02702-z.

## Introduction

Globally, suicide is the fourth leading cause of death in young people [1]. Adolescents are particularly susceptible to self-harm [2], and those presenting to hospital Emergency Departments (EDs) with self-harm are at an elevated risk of suicide [3–5]. Self-harm rates in young people have also been increasing in the United Kingdom (UK) during the last two decades [6]. Identifying young people at risk of engaging in self-harm is imperative since a history of self-harm is one the strongest predictors of suicide [7]. Furthering our understanding of the risk factors for self-harm is essential for identifying high-risk groups and designing preventative interventions for these subgroups [8, 9]. Indeed, addressing common risk factors for suicide and self-harm and creating tailored and targeted interventions for high-risk groups have been set down as priorities in UK national suicide prevention policy [10].

A substantial number of studies have already identified numerous risk factors for self-harm in young people [11]. These risk factors do not happen in isolation and those exposed to one risk factor frequently are exposed to multiple risk factors [12]. As such, a person at an elevated risk of self-harm tend to have a constellation of risk factors (biological, psychological, social, and cultural) in their past [12]. There is also evidence that risk factors cluster into sub-groups [13].

The *Lancet Commission on self-harm (2024)* has mentioned that using latent class analysis (LCA) could be useful in identifying subgroups of self-harm behaviour with different profiles [7]. A previous study applied explorative LCA to the age 13 and 17 waves of the Growing Up in Ireland (GUI) national longitudinal cohort study to identify constellations of risk factors for self-harm at these two ages [13]. ‘Peer problems’, ‘school and substance use problems’, and ‘family conflict and externalising problems’ groups were identified at age 13 and had approximately twice the relative risk of self-harm at age 17 in comparison to the low risk group [13]. Furthermore, our study identified three distinct groups with depression at age 17: one with diagnosed depression and two undiagnosed depression groups with either high or low levels of substance use [13]. In addition, there was a group with low levels of depression and high levels of alcohol use [13].

The identification of these groups could inform the implementation of preventative measures for self-harm, such as anti-bullying or substance use initiatives in schools, which might also positively impact mental health issues in young people [13–15]. However, LCA models may have tendencies to overfitting and a key component of LCA modelling is demonstrating their reproducibility in an external dataset [16]. In addition, the previous study omitted key variables for self-harm in young people, such as childhood abuse and exposure to self-harm, due to the unavailability of these variables in GUI.

The primary aim of this current study was to identify homogenous subgroups in early and late adolescence that exhibit similar risk factors for self-harm, using the Avon Longitudinal Study of Parents and Children (ALSPAC), and to examine the longitudinal associations between the latent classes with the distal outcome of self-harm.

The secondary aim of this study was to conduct cross-cohort comparisons from the results of the current study with those from the previous study that used Irish (or GUI) data [13]. In particular, the current study conducted ‘comparison’ LCAs, that used similar variables to match those used in the previous study [13], and ‘extension’ LCAs, that also included key risk factors (childhood abuse and exposure to self-harm) available in ALSPAC but not in GUI. Cross-cohort comparisons present considerable practical, analytical and conceptual challenges [17]. Data harmonisation is one such example since the risk factors were often measured using different questions or psychometric instruments across the two cohorts [17]. Therefore, having conducted the LCA analyses using ALSPAC, the secondary aim of the current study was to broadly compare the trends between the GUI models from the previous study [13] and their respective ALSPAC comparison and extension models in this current study.

## Methods

### Study design and participants

This current study used data from the Avon Longitudinal Study of Parents and Children (ALSPAC), or ‘Children of the 90s health study’. ALSPAC is a birth cohort study that enrolled *n* = 14,541 pregnant women resident in Avon, UK with expected dates of delivery between 1st April 1991 and 31st December 1992 who were invited to take part in the study [18, 19]. Of the initial pregnancies, there was a total of 14,676 foetuses, resulting in 14,062 live births and 13,988 children who were alive at one year of age [18, 19]. When the oldest children were approximately seven years of age, an attempt was made to bolster the initial sample with eligible cases who had failed to join the study originally [18, 19]. The total sample size was therefore *n* = 15,447 pregnancies, resulting in 15,658 foetuses and of these, *n* = 14,901 children were alive at one year of age [18, 19]. There were *n* = 14,203 unique mothers initially enrolled in the study and, after additional enrolment, there were *n* = 14,833 unique women. Moreover, *n* = 12,113 partners of the mothers have been in contact with the study [18, 19]. Focus clinics were also conducted and were open to all members of the ALSPAC birth cohort [20]. At these clinics, physical and biological measurements, mental health data, and social information were collected from the participants [20].

In the previous study [13], the age 13 wave participants in GUI were aged between 12 and 14 years, with the majority (98%) aged 13 years. For the age 13 comparison and extension models in ALSPAC, the current study included a subset of those who attended “Teen Focus 2” (TF2), a clinical survey of participants at approximately 13.5 years. Specifically, the current study included *n* = 5,407 participants in TF2 aged between 155 months (12.9 years) and 168 months (14 years), inclusive, with the majority (91%) aged 13 years.

The age 17 wave participants in GUI were aged between 16 and 18 years, with the majority (81%) aged 17 years. For the age 17 comparison and extension models in ALSPAC, the current study included a subset of those who attended “Teen Focus 4” (TF4), another clinical survey of participants at approximately 17.5 years. Specifically, the current study included *n* = 5,015 participants in TF4 aged between 192 months (16 years) to 227 months (18.9 years), inclusive, with the majority (77%) aged 17 years.

Adolescence is a period of rapid psychological changes [21] precipitated by neural and social changes in puberty [22]. Hence, while adolescents develop at different rates, it was important to use samples in ALSPAC with similar age distributions to those used in the previous study. Therefore, the age restrictions described hitherto were applied to TF2 and TF4 to make the samples more comparable with the GUI samples. After conducting a bias assessment, there were minimal proportional differences between those included and excluded across the LCA variables. See supplementary materials for further details.

### Exposure variables

An umbrella review of the risk and protective factors for self-harm in young people [11] was used to choose which risk factors were to be included in the LCA models in both this study and the previous study [13]. The full details of how these risk factors were chosen are available in the supplementary materials.

The comparison models, insofar as it was possible, used similar variables in ALSPAC to the variables that were used in the GUI models. The extension models included the same variables as the comparison models but also included child abuse at both ages 13 and 17 and exposure to self-harm at age 17.

Exposure variables for ALSPAC were attained from the Teens Focus surveys, other young person completed surveys, and surveys completed by the mother of the young person both about herself and about the young person. There were also derived variables from ALSPAC, such as social class, that used data from other variables, such as the young person’s mother’s occupation. For the most part, the variables were binary, and in the case when they were continuous, then well-established thresholds were used for dichotomisation. A more detailed description of the risk factors at ages 13 and 17 from the following abridged descriptions can also be viewed in the supplementary material. Please note that the study website contains details of all the data that is available through a fully searchable data dictionary and variable search tool [23].

*Child abuse* and *exposure to self-harm behaviour in others* were reported in ALSPAC but not in GUI and were attained by either reports from the young person or from derived variables.

*Depression/anxiety/stress* at age 13 was measured using the Strengths and Difficulties Questionnaire (SDQ). In particular, the internalising problems sub-score was used for this risk factor, which is the emotional problems sub-score added to the peer problems sub-score [24]. An internalising problems score of 8 or more is considered to be clinically significant and was used as a ‘yes’ for this variable [24].

For *depression/anxiety/stress* at age 17, the revised Clinical Interview Schedule (CIS-R) was used. The CIS-R is a standardised assessment for ‘lay’ interviewers in community, general hospital, occupational and primary care research [25]. There are sub-scores (fatigue, sleep, irritability, anxiety, depression, phobia, etc.) with a separate 0–4 scale [25]. To dichotomise this variable, researchers use a threshold of either two or three and above [25]. This study used a threshold of two or above for each of the depression score and the anxiety score. Hence, a ‘yes’ response to the “depression or anxiety (CIS)” variable meant a participant scored above the threshold in either or both of these scores. Note that in the previous study, for having *depression* at age 17, the Short Moods and Feelings Questionnaire (SMFQ) was used [26]. In particular, a score of eight or above is deemed to be clinically significant [26].

The previous study used a variable in GUI for having been *diagnosed with depression or anxiety* by a doctor, psychiatrist or psychologist at age 17. There was no directly comparable variable in ALSPAC; rather, the “ICD-10 diagnosis or symptoms of depression” was used as a proxy variable for the same. This variable was derived in the ALSPAC dataset using the information provided during the CIS-R.

*Conduct/behavioural/disruptive issues* at ages 13 and 17 was also measured using the SDQ [24]. In particular, the externalising problems sub-score was used for this risk factor, which is the conduct problems sub-score added to the hyperactivity problems sub-score [24]. An externalising problems score of 11 or more is considered significant and was used as a ‘yes’ for this variable [24].

In the previous study, whether the young person had a mental health illness or psychiatric issue was reported by the primary care giver, whereas the Development and Well-Being Assessment (DAWBA) was used in this current study to determine whether the young person had ‘any disorder’ [27]. The DAWBA bands are an ordered-categorical measure of child mental health, where the top two bands are used to indicate ‘present’ and the bottom four bands are used to indicate ‘absent’ [27]. Whether the *parents had a history of mental health problems* was reported by the parents in both studies.

In the previous study’s age 13 GUI model, the Pianta Child-Parent Relationship Scale (CPRS) was used to establish if the young person had *poor family relationships* [28]. Reports from either the young person or the young person’s mother were used for the same risk factor in the age 17 GUI model and in this current study’s ALSPAC models. Similarly, a questionnaire (the Piers Harris popularity sub-score) was used in the previous study’s age 13 GUI model but young person reports were used for *lack of friends or unpopular with peers* for the age 17 GUI model and the current study’s ALSPAC models [29].

Being a *bullying victim*, *substance use*, having *separated or divorced parents*, being involved in *violence*, having a recent *relationship break-up*, *truancy or absence from school*, the young person’s *sexuality*, and *low academic performance* were all reported by the young person or a parent.

In the age 13 ALSPAC extension LCA a combined *substance use* variable was used; whereas for the extension LCA, there were three separate variables for *smoking, alcohol and cannabis use*. There was a variable for other illicit drugs but this was not used since it had a very large amount of missing data. A report of smoking at this age was a report by the young person that they had smoked in the last six months in the Teens Focus 2 (TF2) survey. Using alcohol was determined by a report from the young person that they had drunk alcohol without their parents’ permission in the TF2 survey. Use of cannabis at this age was a report by the young person that they had used cannabis in the TF2 survey. The combined substance use variable was ‘yes’ to smoking, alcohol or cannabis and a ‘no’ response was a no to all these three.

Following on from the age 17 GUI models, there was no variable for combined substance use. *Smoking* at age 17 was a report by the young person that they smoke weekly. For *alcohol use* at age 17, this study used the alcohol use disorders identification test (AUDIT) screening tool [30]. This study used an AUDIT score of ‘hazardous’, ‘harmful’, or ‘high-risk’ as a ‘yes’ for the alcohol use variable and an AUDIT score of ‘low’ for ‘no’ [30]. Using *cannabis* at age 17 meant the young person reported used cannabis 2–4 times a month or more. Finally, *drug use* at age 17 meant the young person had tried using other drugs or substances in the last year.

Both the previous and current studies derived *lower socioeconomic status (SES)* based on the occupations of the parents of the young person. The following SES classes are ranked from highest to lowest: professional workers; managerial and technical; non-manual; skilled manual; semi-skilled; unskilled; all others gainfully occupied; and, unknown; or, validly no social class. An answer of ‘no’ for this variable was used if the young person was from professional workers or managerial and technical households, which are the highest two socioeconomic classes. A ‘yes’ for this variable was if the young person was from non-manual, skilled manual, semi-skilled, or unskilled households. All others not from one these six classes were treated as missing.

### Outcome variables (self-harm outcomes)

In the previous study using GUI, the young person was asked if they had ever hurt themselves on purpose in any way in the last year at ages 17 and age 20 [13]. Similarly, at ages 17 and 20 in ALSPAC, using a combination of young person and mother reports, this current study determined if the young person had hurt themselves on purpose in the last year approximately. This current study also noted the frequency of the self-harm acts.

### Statistical analyses and cross-cohort comparisons

Descriptive statistical analysis was conducted for the self-harm outcomes for the two waves (ages 17 and 20) that contained such data. The methodology used in this current study was largely similar to the previous study [13]. LCAs were conducted at ages 13 and 17 to examine if there were constellations of risk factors for self-harm in early and late adolescence. The large sample sizes (above *n* = 5,000) ensured sufficient statistical power for the LCA models well above the threshold of *n* = 500 suggested by Sinha, Calfee, and Delucchi [16].

Model fit (class numeration) selection was determined using the Bayesian Information Criterion (BIC), since it has been shown to have higher accuracy in comparison to the Akaike information criterion (AIC) and the sample size adjusted BIC (aBIC) [31]. The lowest BIC number was used to determine the number of classes in each model.

The 3-step approach for predicting distal outcomes proposed by Bolck, Croon and Hagenaars (i.e. the BCH 3-step method) to examine longitudinal associations between the LCAs at ages 13 and 17 and self-harm at ages 17 and 20, respectively [32]. In particular, the automatic BCH 3-step approach has been shown by Asparouhov and Muthén to yield identical results to the manual BCH 3-step method when the distal outcome is binary [33].

All data manipulation and cleaning were conducted in the R software. Data were then exported to Mplus where the LCA and distal outcome analysis was conducted [34]. Missing data were handled using full information maximum likelihood (FIML), whereby parameter estimates and standard errors are estimated directly from the observed data by applying iterative computational algorithms to the sample log-likelihood [35–38].

## Results

### Self-harm descriptive statistics

The proportions of participants who self-harmed at age 17 years (9.1%) and at age 20 years (9.4%) can be viewed in Table 1. Out of the proportion of those who had reported that they ever self-harmed by the age of 17 (16.1%), a majority (56.2%) of these participants reported having self-harmed within the last year. Moreover, 71.5% of those who self-harmed at age 17 and 68.4% of those who self-harmed at age 20 (both majorities) exhibited this behaviour more than once in the last 12 months.

**Table 1:** Self-harm descriptive data for age 17 years and age 20 years from ALSPAC

	*n* (%)
**Age 17 years self-harm outcomes (TF4 survey)**	
Lifetime self-harm by age 17 (*n* = 4558)	
Yes	736 (16.1)
No	3,822 (83.9)
Self-harm within last year at age 17 (*n* = 4558)	
Yes	414 (9.1)
No	4,144 (90.9)
Self-harm frequency at age 17 (*n* = 736)	
None	322 (43.8)
Once	118 (16.0)
2–5 times	180 (24.5)
6–10 times	41 (5.6)
More than 10 times	75 (10.2)
**Age 20 years self-harm outcomes**	
Lifetime self-harm by age 20 (*n* = 4269)	
Yes	906 (21.2)
No	3,363 (78.8)
Self-harm within last year at age 20 (*n* = 4269)	
Yes	402 (9.4)
No	3,867 (90.6)
Self-harm frequency at age 20 (*n* = 901)	
None	504 (55.9)
Once	122 (13.5)
2–5 times	167 (18.5)
6–10 times	48 (5.3)
More than 10 times	60 (6.7)

### Latent class model selection

Four-class models were chosen for both the comparison and extension ALSPAC ages 13 and 17 LCAs. The entropy values for the comparison and extension ALSPAC age 13 LCAs were 0.72 and 0.73, respectively, and the entropy value for both the comparison and extension ALSPAC age 17 LCAs was 0.74. See the supplementary material for LCA model information criteria and entropy values. Note that GUI model had an extra class at age 17.

### Age 13 latent classes

**Fig. 1:**
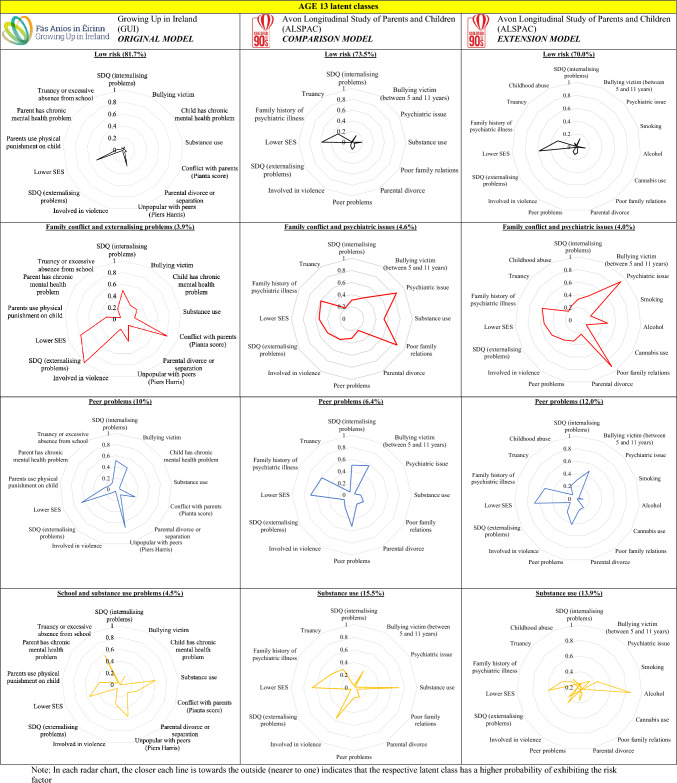
The age 13 latent class models from the GUI study and the respective comparison and extension ALSPAC models. Note: In each radar chart, the closer each line is towards the outside (nearer to one) indicates that the respective latent class has a higher probability of exhibiting the risk factor

Each row in Fig. 1 contains the GUI latent class from the previous study and the corresponding ALSPAC comparison and extension classes.

The majority of participants in the age 13 LCA models were in the ‘low risk’ groups, with a higher majority (approximately 81.7%) in the GUI LCA compared to the comparison and extension models in the ALSPAC LCAs (approximately 73.5% and 70.0%, respectively). These ‘low risk’ groups were characterised by low probabilities for all of the risk factors, except for having lower socioeconomic status, which was broadly similar across the four classes.

The ‘peer problems’ group in the age 13 GUI LCA was characterised by having higher probabilities of being a bullying victim and being unpopular with peers in comparison to the other groups. The comparison group in ALSPAC also had the highest probabilities of being a bullying victim and having peer problems in comparison to the other groups. These GUI and ALSPAC groups had similar approximate sizes of 10% and 6.4%, respectively. Moreover, both of these groups had a moderate probability of having internalising problems. In contrast to the GUI ‘peer problems’ group, which had a low probability of having a parent with a chronic mental health problem, the ALSPAC ‘peer problems’ group had a moderate probability of having a family history of psychiatric illness.

The ‘school and substance use problems’ group (approximately 4.5% of the cohort) in the GUI age 13 LCA was characterised by having the highest probabilities of truancy or excessive absence from school and substance use in comparison to the other groups. This group also had a moderate probability of being unpopular with peers and had the highest probability of being involved in violence compared to the other groups, albeit low in absolute terms. The comparison group in ALSPAC was the ‘substance use’ group, accounting for a comparably higher proportion (15.5%) of its cohort, and was characterised by having a high probability of substance use and higher probabilities of truancy and being involved in violence in comparison to the other groups. Whereas the ‘school and substance use problems’ group in GUI had a moderate probability of being unpopular with peers, the ‘substance use’ group in ALSPAC had a low probability for the same.

The ‘family conflict and externalising problems’ group in the GUI age 13 LCA was approximately 3.9% of the cohort and had high probabilities of having conflict with parents and all this group had externalising problems. Moreover, this group had a higher probability that the young person had a chronic mental health problem in comparison to the three other groups and a moderate probability of having internalising problems as with the ‘peer problems’ group. While the probabilities for having lower socioeconomic status did not differ greatly across the four classes, the probability of exhibiting this risk factor was the highest for the ‘family conflict and externalising problems’ group and lowest for the ‘low risk’ group.

The comparison group in ALSPAC to the ‘family conflict and externalising problems’ group in the GUI age 13 LCA was the ‘family conflict and psychiatric issues’ group, accounting for approximately 4.6% of the cohort. The ‘family and psychiatric issues’ group had high probabilities that the young person had a psychiatric issue and had conflict with their parents. While the ‘family conflict and externalising problems’ group in GUI had low probabilities of substance use and having parents with a chronic mental health problem, the ‘family conflict and psychiatric issues’ group in ALSPAC had moderate probabilities for substance use and having a family history of mental health issues. While all the GUI group had externalising problems and the ALSPAC had a moderate probability for the same, it was much higher compared to the other groups in the ALSPAC comparison model.

In the age 13 extension model in ASLPAC, child abuse had the highest probabilities in the ‘peer problems’ and ‘family and psychiatric issues’ groups, though were still low in absolute terms. Alcohol use without parent’s permission was the main driver for those being identified for substance use as we can see in the ‘substance use’ group. The probability for internalising problems was reduced in the extension ‘peer problems’ group compared to the comparison ‘peer problems’ group.

### Age 17 latent classes

**Fig. 2:**
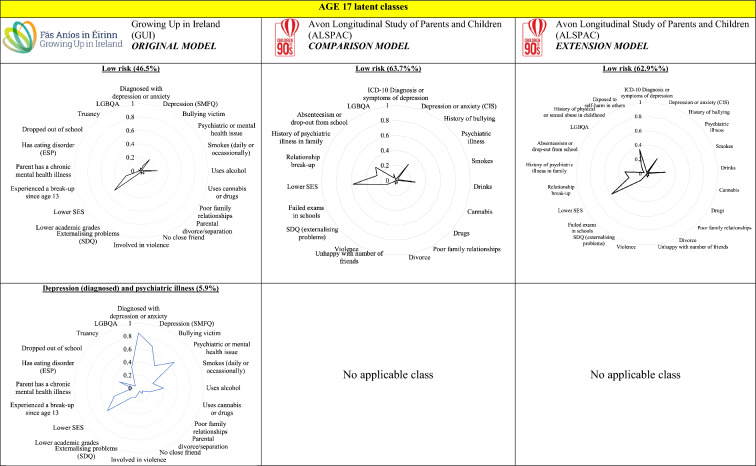

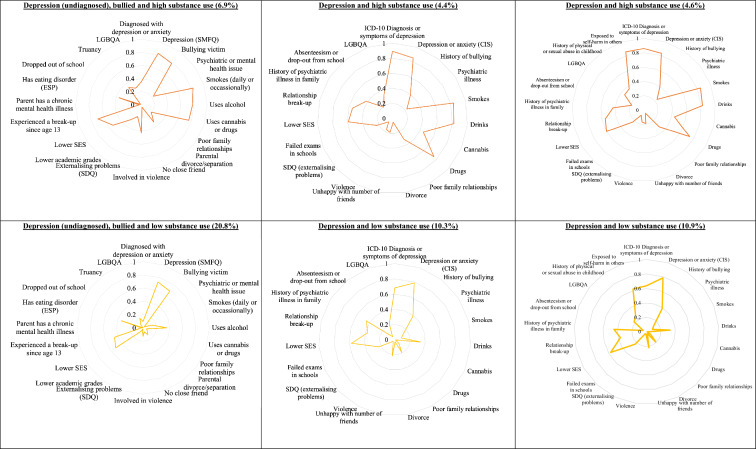

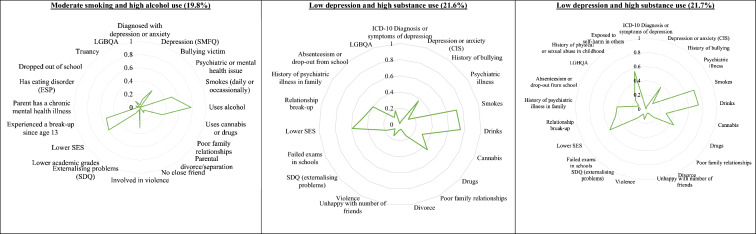
The age 17 latent class models from the GUI study and the respective comparison and extension ALSPAC models. Note: In each radar chart, the closer each line is towards the outside (nearer to one) indicates that the respective latent class has a higher probability of exhibiting the risk factor

The latent classes from the GUI age 17 five-class and ALSPAC age 17 four-class models are displayed in Fig. 2. The extra class in the GUI model may have been due to the omission of the diagnosis of depression by a medical professional in the ASLPAC. Hence the ‘depression (diagnosed) and psychiatric illness’ group in the GUI model, accounting for approximately 5.9% of the cohort, did not have corresponding groups in the ALSPAC models. This group was characterised by having a high probability of being diagnosed with depression by a medical professional as well as a moderate probability of having a psychiatric or mental health issue – the latter risk factor being low in the other four groups. This group also had a high probability of having depression according to the SMFQ instrument but the probability was lower than the other two groups with high undiagnosed depression.

The ‘low risk’ group in the GUI age 17 model had a smaller approximate size (46.5%) compared to the comparison ALSPAC ‘low risk’ group (63.7%). The ‘low risk’ groups in both GUI and ALSPAC age 17 models were characterised by having low probabilities in all risk factors except for having a moderate probability of having lower socioeconomic status.

The ‘depression (undiagnosed), bullied and high substance use’ group in the GUI age 17 LCA and the ‘depression and high substance use’ in the ASLPAC age 17 comparison and extension models had similar sizes of 6.9%, 4.4% and 4.6%, respectively. These groups were characterised by high probabilities of depression and substance use, with moderate-to-high probabilities of experiencing a relationship break-up. The ‘depression (undiagnosed), bullied and high substance use’ group in GUI had a high probability of being a bullying victim whereas the corresponding ALSPAC groups had low levels of the same. The ‘depression and high substance use’ in the ALSPAC extension model also had high probabilities of being exposed to self-harm behaviour in others. While probabilities of previous childhood physical or sexual abuse were low across the groups, it was highest in this group compared to the three other groups.

Similarly, while the ‘depression (undiagnosed), bullied and low substance use’ group in the GUI age 17 LCA had high probabilities of depression and low probabilities of substance use like the corresponding ALSPAC ‘depression and low substance use’ group, the former group had high probabilities of being a bullying victim, whereas the latter had low probabilities in the same.

Finally, the ‘moderate smoking and high alcohol use’ group (about 19.8% of the cohort) in the GUI age 17 model matched the ‘low depression and high substance use’ groups in the ALSPAC age 17 models (about 21.6% of the cohort). These were characterised by higher probabilities of smoking and alcohol use but lower probabilities of using cannabis or drugs and low probabilities of depression.

### Longitudinal associations with self-harm

The longitudinal associations between the age 13 and 17 latent classes and self-harm at ages 17 and 20, respectively, are presented in Table 2. All of the relative risks were in comparison to the low risk groups. Statistically significant results are highlighted in bold.

**Table 2:** Longitudinal associations between latent classes at age 13 and 17 and self-harm at ages 17 and 20, respectively

GUI original model					ALSPAC comparison model				ALSPAC extension model		
Name of latent class	Mean proportion of self-harm at age 17 (95% CI)	*p*-value^α^	Relative Risk	Name of latent class	Mean proportion of self-harm at age 17 (95% CI)	*p*-value^α^	Relative Risk	Name of latent class	Mean proportion of self-harm at age 17 (95% CI)	*p*-value^α^	Relative Risk
*Age 13 LCA models*											
Low risk	9.9% (8.5%, 11.3%)	–	–	Low risk	23.0% (20.8%, 25.2%)	–	–	Low risk	21.3% (18.9%, 23.7%)	–	–
Family conflict and externalising problems	17.1% (6.9%, 27.3%)	0.173	**1.7 (1.3, 2.2)**	Family conflict and psychiatric issues	**51.4% (38.6%, 64.1%)**	** < 0.001**	**2.2 (1.9, 2.5)**	Family conflict and psychiatric issues	**53.2% (40.1%, 66.3%)**	** < 0.001**	**2.5 (2.2, 2.9)**
Peer Problems	**22.6% (14.8%, 30.4%)**	**0.003**	**2.3 (2.0, 2.7)**	Peer problems	**38.0% (24.1%, 51.9%)**	**0.043**	**1.7 (1.4, 1.9)**	Peer problems	**40.0% (29.6%, 50.4%)**	**0.002**	**1.9 (1.7, 2.1)**
School and substance use problems	**22.9% (10.7%, 35.1%)**	**0.041**	**2.3 (1.9, 2.9)**	Substance use	30.6% (21.6%, 39.6%)	0.127	**1.3 (1.2, 1.5)**	Substance use	**32.4% (25.1%, 39.7%)**	**0.006**	**1.5 (1.4, 1.7)**
*Age 17 LCA models*											
Low risk	1.9% (0.5%, 3.2%)	–	–	Low risk	4.0% (2.8%, 5.2%)	–	–	Low risk	3.7% (2.5%, 4.9%)	–	–
Depression (diagnosed) and psychiatric illness	**26.4% (17.6%, 35.2%)**	** < 0.001**	**13.9 (10.2, 19.0)**	*No applicable class*	–	–	–	*No applicable class*	–	–	–
Depression (undiagnosed), bullied and high substance use	**17.9% (9.9%, 25.9%)**	** < 0.001**	**9.4 (6.8, 13.1)**	Depression and high substance use	**30.7 (18.5%, 42.9%)**	** < 0.001**	**7.7 (5.9, 10.0)**	Depression and high substance use	**29.6% (18.4%, 40.8%)**	** < 0.001**	**8.0 (6.1, 10.5)**
Depression (undiagnosed), bullied and low substance use	**14.1% (9.8%, 18.4%)**	** < 0.001**	**7.4 (5.5, 10.0)**	Depression and low substance use	**24.4% (18.3%, 30.5%)**	** < 0.001**	**6.1 (4.9, 7.7)**	Depression and low substance use	**24.7% (18.6%, 30.8%)**	** < 0.001**	**6.7 (5.3, 8.4)**
Moderate smoking and high alcohol use	**7.4% (3.3%, 11.5%)**	**0.023**	**3.9 (2.8, 5.4)**	Low depression and high substance use	**14.5% (10.6%, 18.4%)**	** < 0.001**	**3.6 (2.9, 4.5)**	Low depression and high substance use	**14.5% (10.6%, 18.4%)**	** < 0.001**	**3.9 (3.1, 4.9)**

Notes: ^α^Chi square test of difference in proportion from the low risk reference class; statistical significance is indicated in bold. Relative risk is in comparison to the low risk (reference) class in each LCA

Compared to the low risk classes, the GUI age 13 ‘peer problems’ group had a relative risk of 2.3 (95% CI 2.0–2.7) and the ALSPAC age 13 comparison ‘peer problems’ group had a relative risk of 1.7 (95% CI 1.4–1.9) for self-harm at age 17. There was a higher relative risk in the respective extension model of 1.9 (95% CI 1.7–2.1). While the GUI age 13 ‘school and substance use problems’ group had a statistically significant higher proportion of self-harm at 17 and a relative risk of 2.3 (95% CI 1.9–2.9), the ALSPAC age 13 comparison ‘substance use’ group did not have a statistically significant higher proportion of self-harm at age 17 but its relative risk (1.3 (95% CI 1.2–1.5)) was statistically significant. The same group in the extension model did have a statistically significant higher proportion of self-harm with a relative risk of 1.5 (95% CI 1.4–1.7). While the ‘family conflict and externalising problems’ in the GUI age 13 model did not have a statistically significant higher proportion of self-harm at age 17, compared to the ‘low risk’ group, it did have a statistically significant relative risk of 1.7 (95% CI 1.3–2.2). The ‘family conflict and psychiatric issues’ group in the comparison and extension ALSPAC age 13 models both had statistically significantly higher proportions of self-harm at age 17, with respective relative risks of 2.2 (95% CI 1.9–2.5) and 2.5 (95% CI 2.2–2.9).

The GUI age 17 ‘depression (diagnosed) and psychiatric illness’ group had the highest relative risk (13.9 (95% CI 10.2–19.0)) of self-harm at age 20. There was no comparison group in the ALSPAC cohort. This was followed by the GUI age 17 ‘depression (undiagnosed), bullied and high substance use’ with a relative risk of 9.4 (95% CI 6.8–13.1). Similarly, the ALSPAC age 17 comparison and extension ‘depression and high substance use’ groups had relative risks of 7.7 (95% CI 5.9–10.0) and 8.0 (95% CI 6.1–10.5), respectively.

The GUI age 17 ‘depression (undiagnosed), bullied and low substance use’ had a relative risk of self-harm at age 20 of 7.4 (95% CI 5.5–10.0) and the ALSPAC age 17 comparison and extension ‘depression and low substance use’ had relative risks of 6.1 (95% CI 4.9–7.7) and 6.7 (95% CI 5.3–8.4), respectively. The GUI age 17 ‘moderate smoking and high alcohol use’ had a relative risk of self-harm at age 20 of 3.9 (95% CI 2.8–5.4) and the ALSPAC age 17 comparison and extension ‘low depression and high substance use’ had relative risks of 3.6 (95% CI 2.9–4.5) and 3.9 (95% CI 3.1–4.9), respectively.

## Discussion

The current study used data from the ALSPAC or ‘Children of the 90s health study’, from Avon, UK, to identify subgroups in early and late adolescence with an elevated risk of self-harm. Furthermore, the results from this study were compared to a previous study that used Irish data [13]. The age 13 models from both studies had a low risk group, a peer problems group, and substance use group (with higher levels of truancy and violence) that were similar across the two cohorts. The age 13 family conflict groups were the least similar across the two cohorts, due to data harmonisation issues, and which had high levels of family conflict and at least higher levels of externalising problems and psychiatric or mental health issues compared to the other groups. The age 17 models were very similar across the two cohorts, each with a low risk group, a depression and high substance use group, a depression and low substance use group, and a high alcohol and smoking group. The Irish model had an extra class at age 17 (the ‘depression (diagnosed) and psychiatric illness’ group) due to the omission of the ‘diagnosis of depression by a medical professional’ variable in the ALSPAC dataset at age 17.

While it is not recommended that the results from this study be used in any risk prediction tool for self-harm [39], mental health assessment screening and interventions could potentially be carried out for individuals who present either clinically or in the community with signs of being from these groups. There is no evidence to suggest that asking someone about suicide or self-harm behaviours induce suicidal ideation [40]. Therefore, there may be opportunities for professionals in clinical settings to identify high-risk individuals if they exhibit signals that they are from the high-risk groups in this study. Substance use groups, at an elevated risk for self-harm, have been identified both in early and late adolescence and appropriate referral pathways for mental health assessments and appropriate care could be established since such health risk behaviours are mutually predictive [41]. Even in non-clinical or community settings, opportunistic screening could be utilised by non-healthcare professionals in the community, like teachers, who are often the ones to initially identify self-harm behaviour in adolescents [42] – particularly, since one group at age 13 was defined by school problems.

Examining the school setting is essential in relation to self-harm prevention and intervention measures since it is one of the principal settings that influences the health behaviours, well-being and emotional health of adolescents [43, 44]. Furthermore, many of the risk factors for the high-risk groups, such as bullying or truancy, involve the school setting [11, 13]. Implementing universal school-based programmes for preventing or reducing self-harm and suicide, like Signs of Suicide (SOS) or Youth Aware of Mental Health Programme (YAM), have been found to be successful in reducing suicide attempts [45]. Other measures, like school anti-bullying measures, that target risk factors for self-harm can simultaneously be effective in improving mental health in young people [15]. The school setting has potential for screening, brief interventions and referrals for young people with substance abuse disorders, co-occurring mental health problems, or behavioural issues [46]. Furthermore, social and emotional learning programmes have shown to be successful in improving mental health in adolescents in schools [47].

For young people who have been identified as either potentially at-risk of self-harm, or who have disclosed that they have self-harmed, there needs to be sufficient training, guidelines, procedures and resources in place, since school counsellors are often overloaded [48]. Proper referral pathways need to be put in place to clinical services. Better integration of services and adequate staffing capacity in clinical services is needed to ensure that at-risk individuals can benefit from theses services [7].

Early interventions for adolescents with high undiagnosed psychopathology should also be a priority. The age 17 GUI ‘depression (diagnosed) and psychiatric illness’ group had a slightly lower probability of having depression than the two undiagnosed depression groups in the same model, possibly due to undergoing psychiatric treatment [13]. Undiagnosed individuals may not be receiving the appropriate mental health treatment and, therefore, may be at an increased risk of suicide since the risk of suicide death increases with the number of self-harm episodes [13, 49, 50]. Furthermore, the ‘family conflict and psychiatric issues’ and ‘depression and high substance use’ groups in the extension ALSPAC models at ages 13 and 17, respectively, had the highest probabilities of child abuse compared to the other groups, although the difference was not substantial. Furthermore, the latter group had the highest probability of being exposed to self-harm in either a friend or family member. Both child abuse and exposure to self-harm in others have been shown to be strongly associated with self-harm in adolescents [11].

### Strengths and limitations

The current study used two large rich datasets. Despite the major difficulties and challenges that come with cross-cohort comparisons (in particular, with respect to data harmonisation) [17], the latent classes formed between GUI and ALSPAC were largely similar, thereby demonstrating external validity of the groups in this study. Furthermore, the relative risk figures were very similar across the two studies.

Nevertheless, there were differences in the groups formed and this was most apparent with the age 13 GUI ‘family conflict and externalising problems’ and ALSPAC ‘family conflict and psychiatric issues’ groups. GUI was designed to be nationally representative (using sample weights), whereas the ALSPAC sample may have a degree of selection bias. Data harmonisation was also significant issue for differences between the cohorts. For example, GUI used a response from the primary care giver if the young person had any ongoing chronic physical or mental problem, illness or disability in the form of ‘mental or behavioural disorder’ for the ‘psychiatric issue’ risk factor at age 13. The comparable ALSPAC variable at age 13 used the DAWBA instrument [27].

The entropy from the ALPAC models ranged from 0.72–0.74, slightly below the entropy values of 0.8 for optimal class separation [16, 51]. This was within an acceptable range but suggests that there may be a degree of class uncertainty.

### Further research

The design, implementation and evaluation of targeted interventions for priority groups is part of both British and Irish national mental health policy [10, 52, 53]. The research from this study can help to inform the implementation of these and future policies for addressing mental health in young people. Future research should examine to what extent the recommendations have been implemented, and explore the facilitators and barriers to implementation.

## Conclusion

The *Lancet Commission on self-harm (2024)* mentions that given the complex interplay of factors for self-harm, effective prevention and intervention strategies need to be multifaceted, addressing both the immediate behaviours and the broader psychological and social influences for self-harm [7]. This study endeavoured to use LCA to model the heterogeneity of individuals in respect of the risk factors for self-harm, adding to the research on both immediate behaviours and broader psychological and social influences for self-harm. With respect to immediate behaviours, we know that the majority of adolescents who self-harm do not attend clinical services [54]. Adolescents who exhibit signs of depression or anxiety, have substance use issues, exhibit school and/or peer problems, or who appear to have high levels of family conflict are at a higher risk of self-harm behaviour. Such adolescents could be identified either in clinical or community settings and the appropriate pathways for care need to be place for such individuals. With respect to the broader psychological and social influences for self-harm, the results from this study could inform the design of public health interventions and policy to reduce self-harm behaviour in young people. Examples of such interventions may include implementing universal school-based programmes for preventing or reducing self-harm, school anti-bullying policies, or introducing social and emotional learning programmes.

## Supplementary Information

Below is the link to the electronic supplementary material.

Supplementary file1 (DOCX 159 KB)

## Data Availability

All applications for ALSPAC data must be made to ALSPAC https://www.bristol.ac.uk/alspac/

## Abbreviations

aBIC: Sample sized adjusted Bayesian information criterion
AIC: Akaike information criterion
ALSPAC: Avon Longitudinal Study of Parents and Children
AUDIT: Alcohol use disorders identification test
BCH: Bolck, Croon and Hagenaars (i.e. the BCH 3-step method)
BIC: Bayesian Information criterion
CI: Confidence interval
CIS-R: Revised Clinical Interview Schedule
CPRS: Pianta Child-Parent Relationship Scale
DAWBA: Development and Well-Being Assessment
ED: Emergency Department
ESP: Eating disorder screening tool for primary care
FIML: Full information maximum likelihood
GUI: Growing Up in Ireland
ICD-10: International classification of diseases (version 10)
LCA: Latent class analysis
LGBQA: Lesbian, gay, bisexual, questioning, or asexual
SDQ: Strengths and Difficulties Questionnaire
SES: Socioeconomic status
SMFQ: Short Moods and Feelings Questionnaire
TF2: Teens Focus 2 survey (age ~ 13.5 years)
TF4: Teens Focus 4 survey (age ~ 17.5 years)
UK: United Kingdom

## Code availability

No datasets were generated or analysed during the current study.
