# Supplementary material for: Identifying high-risk groups for self-harm in adolescents using the Avon Longitudinal Study of Parents and Children (ALSPAC): a cross-cohort comparison latent class analysis study
Source: Eur Child Adolesc Psychiatry. 2025 Apr 5;34(9):2843–57. doi: 10.1007/s00787-025-02702-z (PMC12507929; doi:10.1007/s00787-025-02702-z)
Supplement: Supplementary file 1 — Supplementary file1 (DOCX 159 KB) [file 787_2025_2702_MOESM1_ESM.docx]

**Supplementary Materials**

1. GUI exposure variables
2. ALSPAC exposure variables
3. Information criteria
4. Latent class probabilities
5. Bias assessment between those removed in TF2 and TF4
6. References

*Abbreviations*:

aBIC sample sized adjusted Bayesian information criterion

AIC Akaike information criterion

AUDIT Alcohol use disorders identification test

AYA Adolescents and young adults

BCH Bolck, Croon and Hagenaars (i.e. the BCH 3-step method)

BIC Bayesian Information criterion

BPD Borderline personality disorder

CIS-R revised Clinical Interview Schedule

CPRS Pianta Child-Parent Relationship Scale

DAWBA Development and Well-Being Assessment

ESP Eating Disorder Screen for Primary Care

GUI Growing Up in Ireland

ICD-10 International classification of diseases (version 10)

LCA Latent class analysis

LGB Lesbian, gay, bisexual

LGBQA Lesbian, gay, bisexual, questioning, or asexual

NSSI Non-suicidal self-injury

PCG Primary care giver

pOR Pooled odds ratio

SA Suicide attempt

SCG Secondary care giver

SDQ Strengths and Difficulties Questionnaire

SES Socioeconomic status

SMFQ Short Moods and Feelings Questionnaire

TF1 Teens Focus 1 survey (age ~ 12.5 years)

TF2 Teens Focus 2 survey (age ~ 13.5 years)

TF3 Teens Focus 3 survey (age ~ 15.5 years)

TF4 Teens Focus 4 survey (age ~ 17.5 years)

**1. GUI Exposure variables**

An umbrella review of systematic reviews on the risk and protective factors for self-harm in adolescents and young adults (AYAs) was conducted before the precursor Growing Up in Ireland (GUI) study (McEvoy et al., 2023). There were two types of systematic reviews included in this umbrella review: ‘general systematic reviews’ which sought to identify any risk or protective factors for self-harm; and, ‘factor-specific systematic reviews’ that reviewed primary studies examining a specific risk or protective factor, like bullying or impaired sleep for example, for self-harm in young people (McEvoy et al., 2023).

Using these two types of systematic reviews, there were broadly two ways in which the most important risk factors for self-harm in AYAs could be determined (McEvoy et al., 2023). Firstly, the number of times each risk factor was identified by the ‘general reviews’ was tallied and ranked from most to least frequently identified (see Figure 2 from McEvoy et al. 2023) (McEvoy et al., 2023). Secondly, for those systematic reviews that conducted meta-analyses, the sizes of the effect measures (pooled odds ratios - pORs) were examined (McEvoy et al., 2023).

Risk factors were included that were available in the respective wave of the GUI dataset and were either identified by at least three systematic reviews, had a pOR of at least two (see Figure 3 from McEvoy et al. 2023), or indeed satisfied both of these criteria (McEvoy et al., 2023). Given that the umbrella review identified in excess of 60 risk factors, it was necessary to use such criteria to include risk factors in the latent class models for the most important risk factors for self-harm in AYAs. Table S1 provides a list of the risk factors that satisfied one or both of these criteria and information for which of these variables were available in GUI. Being female was not included as a risk factor in the latent class analyses (LCAs) since it was deemed to be a fundamental classifier and the GUI dataset was designed so that it would have an approximately even split of males and females. Instead, the estimated percentages of each sex for each latent class were calculated. The risk factor for being in foster care was also not included since the number of participants for this risk factor was very small (less than 30).

*GUI Risk factors at age 13:*

*Depression/anxiety/stress* was measured using the Strengths and Difficulties Questionnaire (SDQ). In particular, the internalising problems sub-score was used for this risk factor, which is the emotional problems sub-score added to the peer problems sub-score (Goodman and Goodman, 2009). An internalising problems score of 8 or more is considered to be clinically significant and was used as a ‘yes’ for this variable (Goodman and Goodman, 2009).

*Bullying* was reported by the primary care giver (PCG) that the young person had been bullied in the last three months. *Other psychiatric illness* was a report by the PCG of ‘yes’ to the question ‘Does child have any ongoing chronic physical or mental problem, illness or disability?’ and that the form of this was a ‘mental and behavioural disorder’ based on the WHO International Classification of Diseases (ICD-10) (World Health Organization, 2004). For *family history of mental health problems*, this question was also asked of the PCG and the secondary care giver (SCG). A ‘yes’ response to this variable was if one or both care givers answered ‘yes’.

For *substance use or abuse*, the young person had to answer ‘yes’ to at least one of smoked cigarettes at least once a week; consumed alcohol at least once a month; or, ever tried using cannabis, sniffing glues or illicit drugs. Since these variables were less common at age 13 compared to at age 17, these were pooled into one variable at age 13 but kept separate at age 17.

For *poor family relationships*, this study used a derived variable in the GUI dataset that used the Pianta Child-Parent Relationship Scale (CPRS), a self-report instrument completed by mothers and fathers that assesses parents’ perceptions of their relationships with their sons and daughters (Pianta, 2011). The mean score for the PCG was 15.1 and the standard deviation was 6.4 so two standard deviations above the mean was 27.9. The mean score for the secondary care giver was 15.0 and the standard deviation was 5.8 so two standard deviations above the means was 26.6. Since an established threshold score for this variable was not found, ‘yes’ was defined for this variable if the young person’s CPRS score was two standard deviations above the mean in the primary or secondary care giver score in either or both of these.

*Parental divorce/separation* was obtained by a direct report from the primary care giver. For *lack of friends or being unpopular*, we used the Piers-Harris Children’s Self-Concept popularity sub-scale (Community-University Partnership for the Study of Children Youth and Families, 2011). This is one of six subscales in the Piers-Harris Children’s Self-Concept Scale (Community-University Partnership for the Study of Children Youth and Families, 2011). For each of the subscales, a score of ≤29 is considered very low, 30-39 is low, 40-44 is low average, 45-55 is average and >56 is above average (Community-University Partnership for the Study of Children Youth and Families, 2011). This was a derived variable in the GUI dataset and ‘yes’ referred to ‘very low’ or ‘low’ in this subscale.

*Violence or peer conflict (involved in)* was a report by the young person that they had been involved in a serious physical fight where someone got badly hurt or needed to see a doctor. *Conduct/behavioural/disruptive issues* was measured using the SDQ (Goodman and Goodman, 2009). In particular, the externalising problems sub-score was used for this risk factor, which is the conduct problems sub-score added to the hyperactivity problems sub-score (Goodman and Goodman, 2009). An externalising problems score of 11 or more is considered significant and was used as a ‘yes’ for this variable (Goodman and Goodman, 2009).

This study used a GUI derived variable for *lower socioeconomic status (SES)*. GUI works out the young person’s household SES class based on the professions of the primary and secondary care givers. The following SES classes are ranked from highest to lowest: professional workers; managerial and technical; non-manual; skilled manual; semi-skilled; unskilled; all others gainfully occupied; and, unknown; or, validly no social class. An answer of ‘no’ for this variable was used if the young person was from professional workers or managerial and technical households, which are the highest two socioeconomic classes. A ‘yes’ for this variable was if the young person was from non-manual, skilled manual, semi-skilled, or unskilled households. All others not from one these six classes were treated as missing.

For *harsh/controlling parent*, a report by the young person that the parent uses physical punishment sometimes or always was used. For *school truancy or excessive absence from school*, this variable was recorded as ‘yes’ if either the young person was absent from school for more than 20 days or answered that they occasionally, quite often or always skipped classes or mitched.

*GUI Risk factors at age 17:*

*Diagnosed with depression or anxiety* was determined by a ‘yes’ response by the young person when asked if they had been diagnosed with depression or anxiety by a doctor, psychiatrist, or psychologist. For having *depression*, which may or may not have been diagnosed, we used the Short Moods and Feelings Questionnaire (SMFQ) (Angold, 1995). In particular, a score of eight or above is deemed to be clinically significant (Angold, 1995).

Being a *bullying victim* was determined by a report by the young person that they had experienced some form of bullying (physical, verbal, exclusion etc.) in the last three months. Whether the young person had a *psychiatric or mental health issue* was determined by a report from the primary care giver that the young person had a psychological or emotional condition.

With respect to the substance use variables, *smoking* denoted that the young person responded as smoking daily or occasionally. *Uses alcohol* denoted that the young person responded that they consumed alcohol two to four times a month, two to three times a week or four or more times a week. *Uses cannabis or drugs* was a ‘yes’ response by the young person that they use cannabis occasionally or more than once a week or tried using illicit drugs (such as ecstasy, cocaine, or heroin).

For *poor family relationships*, the young person was asked to rate how well members of their household got on from one to ten, with one being ‘we don’t get on at all’ and ten being ‘we get on very well’. Having poor family relationships was deemed to be significant in this study if the young person recorded below four for this question. *Parental divorce or separation* was determined by a response by the young person that this had occurred since the young person was aged 13 (i.e. the recording of the last wave). Having *no close friend* was also determined by a report from the young person.

Been *involved in violence* was a report by the young person that in the last year they had hit, kicked or punched someone in order to hurt or injure them, or had been involved in a serious fight where someone got badly hurt or needed to see a doctor. *Externalising problems* was measured using the SDQ, like at age 13, with a score of 11 or more considered significant (Goodman and Goodman, 2009).

The *lower academic grades* risk factor was determined by a subjective report by the young person that they did just below average or below average in tests or exams in comparison to other people their age. *Not from a professional or managerial SES* was determined in the same way as for age 13.

*Experienced a break-up since the age of 13* was reported by the young person. Whether the *primary or secondary care giver had a chronic mental health illness* was determined in the same way as for age 13. For the young person having an *eating disorder*, the Eating Disorder Screen for Primary Care (ESP) was used, in which a score of 3 or more indicates a positive screen for an eating disorder (Cotton et al., 2003).

*Dropped out of secondary school early* (without completing the Leaving Certificate – the state examination that most young people complete prior to finishing school); mitching or *truanting* from school; and, being lesbian, gay bisexual, questioning their sexuality or being asexual (*LGBQA*) were all self-reported by the young person.

| **Table S1: Risk factor indicator variables used in the GUI latent class analyses at ages 13 and 17** | | | | | | | | |
| --- | --- | --- | --- | --- | --- | --- | --- | --- |
| Risk factor | Identified by N systematic reviews ^γ^ | pOR ^γ^  (95% CI) (Reference) | Available in GUI wave 2 (age 13) | Number of variables used for this risk factor in GUI wave 2 (age 13) | GUI wave 2 (age 13) | Available in GUI wave 3 (age 17) | Number of variables used for this risk factor in GUI wave 3 (age 17) | GUI wave 3 (age 17) |
| Childhood neglect/(sexual) abuse | 9 | 4.1 (2.3–7.3) (Zatti et al., 2017) | 🗶 | 0 | --- | 🗶 | 0 | --- |
| Depression/anxiety/stress | 8 | 6.6 (2.3-18.9) (Pozuelo et al., 2021) | ✓ | 1 | High SDQ internalising problems | ✓ | 2 | Depression (SMFQ) & Diagnosed with depression or anxiety by a doctor, psychiatrist or psychologist |
| Bullying or cyberbullying | 7 | 6.3 (1.5–25.9) (Miranda-Mendizabal et al., 2019) | ✓ | 1 | Bullying victim | ✓ | 1 | Bullying victim |
| ACEs/ALEs/Trauma (general) | 7 | 3.0 (1.3–6.6) (Miranda-Mendizabal et al., 2019) | 🗶 | 0 | --- | 🗶 | 0 | --- |
| Other psychiatric illness | 7 | 4.2 (3.3–5.5) (Miranda-Mendizabal et al., 2019) | ✓ | 1 | Young person has an on-going chronic mental or behavioural disorder | ✓ | 1 | Young person has psychiatric or mental health issue |
| Substance use or abuse | 6 | 4.4 (2.5–7.8) (Miranda-Mendizabal et al., 2019) | ✓ | 1 | Substance use/abuse (smoking, alcohol or drugs) | ✓ | 3 | Smokes (daily or occasionally), uses alcohol & uses cannabis or illicit drugs |
| Poor family relationships | 6 | --- | ✓ | 1 | High conflict with parents (Pianta score) | ✓ | 1 | Low level of perceived family relationships |
| Parental divorce/separation | 6 | 2.1 (1.1–4.1) (Zatti et al., 2017) | ✓ | 1 | Parents are separated or divorced | ✓ | 1 | Parents are separated or divorced |
| Lack of friends or being unpopular | 5 | 1.1 (1.03–1.2) (Miranda-Mendizabal et al., 2019) | ✓ | 1 | Low Piers Harris popularity score | ✓ | 1 | Young person has no close friend(s) |
| Exposure to NSSI/SA in others | 5 | 3.7 (3.5-4.0) (Geulayov et al., 2012) | 🗶 | 0 | --- | 🗶 | 0 | --- |
| Violence or peer conflict (involved in) | 4 | 1.8 (1.5–2.3) (Miranda-Mendizabal et al., 2019) | ✓ | 1 | Involved in violence | ✓ | 1 | Involved in violence |
| Emotional distress/disorder | 4 | --- | ✓ | 1 | High SDQ internalising problems (already listed) | ✓ | 1 | Depression (SMFQ) (already listed) |
| Conduct/behavioural/disruptive issues | 4 | 8.8 (2.8–27.8) (Miranda-Mendizabal et al., 2019) | ✓ | 1 | High SDQ externalising problems | ✓ | 1 | High SDQ externalising problems |
| Personality disorder or BPD | 4 | 7.9 (3.8–16.4) (Miranda-Mendizabal et al., 2019) | 🗶 | 0 | --- | 🗶 | 0 | --- |
| Poor academic performance | 3 | 1.5 (1.2–1.8) (Castellví et al., 2020) | 🗶 | 0 | --- | ✓ | 1 | Young person perceives they score below average compared to their peers in tests |
| Low SES (real or perceived) | 3 | --- | ✓ | 1 | Not from a professional or managerial family household | ✓ | 1 | Not from a professional or managerial family household |
| Harsh/controlling parent (perceived) | 3 | --- | ✓ | 1 | Parent uses physical punishment on young person | 🗶 | 0 | --- |
| Relationship problems/breakup | 3 | --- | 🗶 | 0 | --- | ✓ | 1 | Young person experienced a break-up since age 13 |
| Being female | 3 | 2.0 (1.5-2.5) (Miranda-Mendizabal et al., 2019) | ✓ ^α^ | 0 | --- | ✓ (not used in LCA) | 0 | --- |
| Impulsiveness | 3 | --- | 🗶 | 0 | --- | 🗶 | 0 | --- |
| Hopelessness/pessimism | 3 | 1.7 (1.04–2.9) (Miranda-Mendizabal et al., 2019) | 🗶 | 0 | --- | 🗶 | 0 | --- |
| Family history of mental health problems | 3 | 2.63 (2.0–3.5) (Miranda-Mendizabal et al., 2019) | ✓ | 1 | Parent has an on-going chronic mental or behavioural disorder | ✓ | 1 | Parent has an on-going chronic mental or behavioural disorder |
| Previous self-harm/SA behaviour | 3 | 31.3 (9.4–104.8) (Miranda-Mendizabal et al., 2019) | 🗶 | 0 | --- | 🗶 | 0 | --- |
| Relationship victim | 3 | 2.4 (1.4–4.0) (Cheek et al., 2020) | 🗶 | 0 | --- | 🗶 | 0 | --- |
| School truancy/drop-out | 2 | 6.4 (3.0–13.7) (Castellví et al., 2020) | ✓ | 1 | Truancy or excessive absence from school | ✓ | 2 | Dropped out of school (without completing the Leaving Certificate) & truancy |
| Eating disorders | 1 | 5.3 (2.0–13.6) (Miranda-Mendizabal et al., 2019) | 🗶 | 0 | --- | ✓ | 1 | Eating disorder (ESP) |
| Suicide of friend/family | 1 | 3.3 (3.1-3.5) (Geulayov et al., 2012) | 🗶 | 0 | --- | 🗶 | 0 | --- |
| Low self-esteem | 1 | 2.0 (1.4-2.9) (Soto-Sanz et al., 2019) | 🗶 | 0 | --- | 🗶 | 0 | --- |
| LGB | 1 | 3.0 (2.5–3.7) (Batejan et al., 2015) | 🗶 | 0 | --- | ✓ | 1 | LGBQA |
| Foster Care | 1 | 3.9 (3.1-4.8) (Evans et al., 2017) | ✓ ^β^ | 0 | --- | ✓ (not used in LCA) | 0 | --- |

Notes: ^α^ Being female was not included in the LCA models – instead an estimated proportion of females was calculated for each LCA group; ^β^ Being in foster care was also not used in the LCA models due to very small numbers of participants with this risk factor in the GUI dataset; γ For further information for the number of times a risk factor was identified by ‘general reviews’ and pORs, please see McEvoy et al 2023 (McEvoy et al., 2023); please see abbreviation section

**2. ALSPAC Exposure variables**

Table S2 lists the risk factors used in both the age 13 and age 17 LCAs for ALSPAC. These were chosen using the same method as in the precursor study (see section 1).

*ALSPAC Risk factors at age 13:*

*Depression/anxiety/stress* was measured using the SDQ, in the same way as GUI, and was reported by the mother of the young person at age 13. An internalising problems score of 8 or more is considered to be clinically significant and was used as a ‘yes’ for this variable (Goodman and Goodman, 2009).

*Conduct/behavioural/disruptive issues* was also measured using the SDQ at age 13 (Goodman and Goodman, 2009). An externalising problems score of 11 or more is considered significant and was used as a ‘yes’ for this variable (Goodman and Goodman, 2009).

Being a *bullying victim* between the ages of five to eleven was determined from a derived variable from ALSPAC.

The Development and Well-Being Assessment (DAWBA), reported by the mother of the young person, was used to determine whether the young person had a *psychiatric illness*. The DAWBA bands are an ordered-categorical measure of child mental health, where the top two bands are used to indicate 'present' and the bottom four bands are used to indicate 'absent' (Goodman et al., 2011). Any disorder present according to the DAWBA was used to signify that the young person had a psychiatric disorder or illness.

In the age 13 ALSPAC extension LCA a combined *substance use* variable was used; whereas for the extension LCA, there were three separate variables for *smoking, alcohol and cannabis use*. There was a variable for other illicit drugs but this was not used since it had a very large amount of missing data. A report of smoking at this age was a report by the young person that they had smoked in the last six months in the Teens Focus 2 (TF2) survey. Using alcohol was determined by a report from the young person that they had drunk alcohol without their parents’ permission in the TF2 survey. Use of cannabis at this age was a report by the young person that they had used cannabis in the TF2 survey. The combined substance use variable was ‘yes’ to smoking, alcohol or cannabis and a ‘no’ response was a no to all these three.

*Poor family relations* or conflict with one’s family was a response of ‘quite a lot’ or ‘a great deal’ to any of the following statements reported by the mother of the young person:

- Degree to which separation worries from person attached to interfered with child's day-to-day life with respondent/rest of family
- Degree to which problems interfered how well child gets on with respondent/rest of family in day-to-day life
- Degree to which acts/thoughts have interfered how well child gets on with respondent/rest of family
- Degree to which worries interfered how well child gets on with respondent/rest of family in day-to-day life
- Degree to which being upset/distressed interfered how well child gets on with respondent/rest of family in day-to-day life
- Degree to which difficulties interfered how well child gets on with respondent/rest of family in day-to-day life
- Degree to which awkward/troublesome behaviour interfered how well child gets on with respondent/rest of family in day-to-day life
- Frequency child's behaviour often disrupts normal family life over last 6 months

*Divorce of parents* before the age of 11 was determined by a report from the young person retrospectively at age 27 years approximately.

*Problems with peers* was self-reported by the young person at age 13 that school was not a place where they felt others accepted them, was a place where they felt lonely, or, was a place where they did not get on with other pupils their age.

Being *involved in violence* was reported in the TF1 survey (at age 12.5 approximately), which the majority of people in the TF2 survey also completed. In particular, this was a ‘yes’ response that the young person had beaten someone up or got into fights.

Similar to GUI, this study used a ASLPAC derived variable for SES. ALSPAC derived the young person’s household SES class based on the professions of the mother. The variable ‘not from a professional or managerial class’ was the same as for GUI.

A *history of family mental illness* was determined by:

- a yes response by the mother that she had mental health problems in the last two years when the child was aged 12, or,
- a yes response by the mother that she ever had bulimia, alcoholism, schizophrenia, anorexia nervosa, severe depression, or other psychiatric problems when the child was aged 11, or,
- or a report that a parent had a mental illness before the child was aged 11

*Truancy* was determined from the TF1 survey where the young person was asked if they had ever engaged in truancy from school.

*Childhood abuse* was determined using a derived variable that the young person endured physical or sexual abuse

Note that variables for eating disorders and previous suicide attempt of mother was not used to very small numbers (<30).

The variable for ‘dating violence’ was also excluded since it was deemed inappropriate for the younger AYA range and was covered to some degree under being a bullying victim.

*ASLPAC Risk factors at age 17:*

There were two variables used for *depression or anxiety* of the young person. The revised Clinical Interview Schedule (CIS-R) is a standardised assessment for ‘lay’ interviewers in community, general hospital, occupational and primary care research (Lewis et al., 1992). There are sub-scores (fatigue, sleep, irritability, anxiety, depression, phobia, etc.) with a separate 0-4 scale (Lewis et al., 1992). To dichotomise this variable, researchers use a threshold of either two or three and above (Lewis et al., 1992). This study used a threshold of two or above for each of the depression score and the anxiety score. Hence, a ‘yes’ response to the “depression or anxiety (CIS)” variable meant a participant scored above the threshold in either or both of these scores. The “ICD-10 diagnosis or symptoms of depression” was used as a proxy variable for the GUI variable ‘diagnosed by depression’. This variable was derived in the ALSPAC dataset using the information provided during the CIS. This response was ‘yes’ if according to the information that the participant had given, they had met the ICD-10 criteria for having diagnosis or symptoms of depression.

Being a *bullying victim* was a derived variable that determined if the participant had experienced bullying up to the age of 17.

Having a *psychiatric illness* was determined from those in TF4 who also completed TF3 and completed the DAWBA assessment in the same way as for the age 13 cohort.

Following on from the GUI models, there was no variable for combined substance use. *Smoking* was a report by the young person that they smoke weekly. For *alcohol use*, this study used the alcohol use disorders identification test (AUDIT) screening tool (Babor et al., 2001). This study used an AUDIT score of ‘hazardous’, ‘harmful’, or ‘high-risk’ as a ‘yes’ for the alcohol use variable and an AUDIT score of ‘low’ for ‘no’ (Babor et al., 2001). Using *cannabis* meant the young person reported used cannabis 2-4 times a month or more. Finally. *drug use* meant the young person had tried using other drugs or substances in the last year.

For *poor family relationships*, the young person replied that during the last few months, they had replied ‘not very close’ or ‘not close at all” to how well they had been getting on with their family. *Separation or divorce* was reported by the young person to have occurred in the last year. The young person also reported that they were ‘quite unhappy’ or ‘unhappy’ with the number of friends that they had or had no friends for the *unpopular* variable.

For being *involved in violence*, the young person replied that they had hit, kicked, punched or attacked someone with the intention of really hurting them.

For *low academic achievement*, the young person replied that they had experienced failure in end of year exams at school.

For *conduct or behavioural issues*, this study used an earlier report of the SDQ externalising problems score by the mother but still had to be in the 16-18 years age range (inclusive). This score was measured in the same aforementioned method as above.

*Lower socioeconomic status* was measured in the same way was for age 13.

The young person reported that they had experienced a *break-up* from a boyfriend or girlfriend in the last year.

A family *history of mental illness* was determined the same way as for age 13. Updated variables were created to account for updated mental illnesses in the same until age 17 but these resulted in high levels of missing data and so were not used.

*High absenteeism or drop-out* from school was determined by an earlier report (but still had to be in the 16-18 years age range (inclusive)) from the young person that they attended school 60% of time or lower or were no longer registered at school.

Being *LGBQA* was a report by the young person that they were not heterosexual from the TF3 survey but still had to be in the 16-18 years age range (inclusive).

*Childhood abuse* was a derived variable of young person’s report of physical or sexual abuse up to the age of 16.

*Exposure to self-harm* was a report by the young person that they had been exposed to self-harm by family or friends.

| **Table S2: Risk factor indicator variables used in the ALSPAC latent class analyses at ages 13 and 17** | | | | | | | | |
| --- | --- | --- | --- | --- | --- | --- | --- | --- |
| Risk factor | Identified by N systematic reviews ^γ^ | pOR ^γ^  (95% CI) (Reference) | Available in ALSPAC (age 13) | Number of variables used for this risk factor in ALSPAC (age 13) | ALSPAC (age 13)  Participants in TF2 | Available in ALSPAC (age 17) | Number of variables used for this risk factor in ALSPAC (age 17) | ALSPAC (age 17)  Participants in TF4 |
| Childhood neglect/(sexual) abuse | 9 | 4.1 (2.3–7.3) (Zatti et al., 2017) | ✓ | 1 | Derived variable of physical or sexual abuse between the ages of 5-11 years | ✓ | 1 | Sexual abuse up to 11 or physical abuse to age 16 |
| Depression/anxiety/stress | 8 | 6.6 (2.3-18.9) (Pozuelo et al., 2021) | ✓ | 1 | High SDQ internalising problems (reported by mother at age 13) | ✓ | 2 | TF4 CIS-R depression or anxiety score  TF4 Depression (ICD-10 diagnosis or symptoms) |
| Bullying or cyberbullying | 7 | 6.3 (1.5–25.9) (Miranda-Mendizabal et al., 2019) | ✓ | 1 | Bullying victim between the ages of 5 to 11 (derived variable in ALSPAC) | ✓ | 1 | History of bullying up to age 17 |
| ACEs/ALEs/Trauma (general) | 7 | 3.0 (1.3–6.6) (Miranda-Mendizabal et al., 2019) | 🗶 | 0 | Covered in childhood abuse | 🗶 | 0 | Covered in childhood abuse |
| Other psychiatric illness | 7 | 4.2 (3.3–5.5) (Miranda-Mendizabal et al., 2019) | ✓ | 1 | Age 13 DAWBA Any psychiatric disorder (reported by the mother at age 13) | ✓ | 1 | TF3 DAWBA Any psychiatric disorder |
| Substance use or abuse | 6 | 4.4 (2.5–7.8) (Miranda-Mendizabal et al., 2019) | ✓ | 4 | 1. Young person has smoked cigarettes in the last six months  2. Young person has consumed alcohol without parents’ permission  3. Young person tried cannabis  4. Combined substance use variable  (Self-reported TF2 survey) | ✓ | 4 | TF4 Smokes weekly  TF4 AUDIT alcohol use: ‘hazardous’, ‘harmful’, or ‘high-risk’  TF4 Uses cannabis 2-4 times monthly or more  TF4 Tried illicit drugs in the last year |
| Poor family relationships | 6 | --- | ✓ | 1 | The actions or thoughts of the young person interferes with how they get on with the rest of the family (reported by the mother of young person) | ✓ | 1 | TF4 Does not get on with family |
| Parental divorce/separation | 6 | 2.1 (1.1–4.1) (Zatti et al., 2017) | ✓ | 1 | Divorce or separation of parents before 11 (reported by the young person at 27 years) | ✓ | 1 | Separation or divorce of parents in the last year |
| Lack of friends or being unpopular | 5 | 1.1 (1.03–1.2) (Miranda-Mendizabal et al., 2019) | ✓ | 1 | Peer problems at school (school is a place where pupils don’t accept them; a place they feel lonely; or, do not get on with other pupils) (self-report age 13) | ✓ | 1 | TF4: Unhappy with no friends or has no friends |
| Exposure to NSSI/SA in others | 5 | 3.7 (3.5-4.0) (Geulayov et al., 2012) | 🗶 | 0 | --- | ✓ | 1 | At age 16, YP exposed to SH in family/friend |
| Violence or peer conflict (involved in) | 4 | 1.8 (1.5–2.3) (Miranda-Mendizabal et al., 2019) | ✓ | 1 | Teenager has beaten someone up/got into fights (self-report in TF1) | ✓ | 1 | TF4: involved in violence |
| Emotional distress/disorder | 4 | --- | 🗶 | 0 | Covered in depression/anxiety and/or psychiatric illness | 🗶 | 0 | Covered in depression/anxiety and/or psychiatric illness |
| Conduct/behavioural/disruptive issues | 4 | 8.8 (2.8–27.8) (Miranda-Mendizabal et al., 2019) | ✓ | 1 | High SDQ externalising problems (reported by the mother at age 13) | ✓ | 1 | AGE 16: SDQ externalising problems |
| Personality disorder or BPD | 4 | 7.9 (3.8–16.4) (Miranda-Mendizabal et al., 2019) | 🗶 | 0 | --- | 🗶 | 0 | --- |
| Poor academic performance | 3 | 1.5 (1.2–1.8) (Castellví et al., 2020) | 🗶 | 0 | --- | ✓ | 1 | TF4: Failed end of year exams |
| Low SES (real or perceived) | 3 | --- | ✓ | 1 | Not from a professional or managerial social class based on occupation of mother (derived variable in ALSPAC) | ✓ | 1 | Not from a professional or managerial social class based on occupation of mother |
| Harsh/controlling parent (perceived) | 3 | --- | 🗶 | 0 | --- | 🗶 | 0 | --- |
| Relationship problems/breakup | 3 | --- | 🗶 | 0 | --- | ✓ | 1 | TF4: Breakup in the last year |
| Being female | 3 | 2.0 (1.5-2.5) (Miranda-Mendizabal et al., 2019) | ✓ (not used in LCA) ^α^ | 1 | Being female | ✓ (not used in LCA) | 1 | Being female |
| Impulsiveness | 3 | --- | 🗶 | 0 | --- | 🗶 | 0 | --- |
| Hopelessness/pessimism | 3 | 1.7 (1.04–2.9) (Miranda-Mendizabal et al., 2019) | 🗶 | 0 | Covered under depression and anxiety | 🗶 | 0 | Covered under depression and anxiety |
| Family history of mental health problems | 3 | 2.63 (2.0–3.5) (Miranda-Mendizabal et al., 2019) | ✓ | 1 | Mother ever had a psychiatric illness by time child was age 11, or  Mother had mental health problem in last two years (child age 12), or  Parent mental illness before age 11  (Parent reported) | ✓ | 1 | Mother ever had a psychiatric illness by time child was age 11, or  Mother had mental health problem in last two years (child age 12), or  Parent mental illness before age 11 |
| Previous self-harm/SA behaviour | 3 | 31.3 (9.4–104.8) (Miranda-Mendizabal et al., 2019) | ✓ (not used in LCA) | 0 | Not to be used – exposure same as outcome | 🗶 | 0 | Not to be used – exposure same as outcome |
| Relationship victim | 3 | 2.4 (1.4–4.0) (Cheek et al., 2020) | ✓ (not used in LCA) ^δ^ | 1 | Teenager a victim of some dating violence: TF2 (NOT USED) | 🗶 | 0 | --- |
| School truancy/drop-out | 2 | 6.4 (3.0–13.7) (Castellví et al., 2020) | ✓ | 1 | Teenager has played truant (young person reported in TF1) | ✓ | 1 | Over 40% absenteeism or drop-out |
| Eating disorders | 1 | 5.3 (2.0–13.6) (Miranda-Mendizabal et al., 2019) | ✓ (not used in LCA) ^β^ | 2 | Eating disorder report by parent or doctor at age 13  Eating disorder (DAWBA)  Too small numbers | ✓ (not used in LCA) | 2 | Treated for an eating disorder at 16 (SMALL)  Treated for an eating disorder at 18 (SMALL)  Too small numbers |
| Suicide of friend/family | 1 | 3.3 (3.1-3.5) (Geulayov et al., 2012) | ✓ (not used in LCA) ^β^ | 1 | Previous suicide attempt by mother by age 11  Too small numbers | 🗶 | 0 | --- |
| Low self-esteem | 1 | 2.0 (1.4-2.9) (Soto-Sanz et al., 2019) | 🗶 | 0 | --- | ✓ (not used in LCA) | 1 | Bottom 10^th^ percentile of Bachman scale |
| LGB | 1 | 3.0 (2.5–3.7) (Batejan et al., 2015) | 🗶 | 0 | --- | ✓ | 1 | LGBQA TF3 |
| Foster Care | 1 | 3.9 (3.1-4.8) (Evans et al., 2017) | 🗶 | 0 | --- | 🗶 | 0 | --- |

Notes: ^α^ Being female was not included in the LCA models – instead an estimated proportion of females was calculated for each LCA group; ^β^ Eating disorder and suicide attempt of mother not used in the LCA models due to very small numbers of participants with this risk factor in the ALSPAC dataset; γ For further information for the number of times a risk factor was identified by ‘general reviews’ and pORs, please see McEvoy et al 2023 (McEvoy et al., 2023); ^δ^ not used since inappropriate for this age group; please see abbreviation section

**3. Information criteria**

| **Table S3: Model fit statistics and entropy for the GUI latent class analysis at ages 13 and 17** | | | | |
| --- | --- | --- | --- | --- |
|  | **AIC** | **BIC** | **Sample size adjusted BIC** | **Entropy** |
|  |  |  |  |  |
| **Age 13 classes** |  |  |  |  |
| 1 | 59139.9 | 59229.9 | 59188.6 | N/A |
| 2 | 56715.7 | 56902.7 | 56816.9 | 0.76 |
| 3 | 56428.5 | 56712.5 | 56582.2 | 0.81 |
| 4 | 56242.5 | **56623.5** | 56448.7 | 0.83 |
| 5 | 56150.0 | 56627.9 | 56408.7 | 0.81 |
| 6 | 56082.3 | 56657.1 | **56393.4** | 0.61 |
| 7 | **56032** | 56703.8 | 56395.6 | 0.61 |
|  |  |  |  |  |
| **Age 17 classes** |  |  |  |  |
| 1 | 91064.8 | 91199.5 | 91135.9 | N/A |
| 2 | 87175.0 | 87451.1 | 87320.8 | 0.67 |
| 3 | 86208.3 | 86625.8 | 86428.8 | 0.70 |
| 4 | 85617.7 | 86176.7 | 85913.0 | 0.65 |
| 5 | 85259.5 | 85959.9 | 85629.4 | 0.68 |
| 6 | 85093.0 | **85934.9** | 85537.7 | 0.70 |
| 7 | **84953.9** | 85937.1 | **85473.2** | 0.73 |
|  |  |  |  |  |

| **Table S4: Model fit statistics and entropy for the ALSPAC latent class analysis at ages 13 and 17** | | | | |
| --- | --- | --- | --- | --- |
|  | **AIC** | **BIC** | **Sample size adjusted BIC** | **Entropy** |
|  |  |  |  |  |
| **Comparable. model** |  |  |  |  |
| **Age 13 classes** |  |  |  |  |
| 1 | 44994.3 | 45073.4 | 45035.3 | N/A |
| 2 | 43453.5 | 43618.4 | 43538.9 | 0.75 |
| 3 | 42998.2 | 43248.8 | 43128.1 | 0.70 |
| 4 | 42868.5 | **43204.9** | **43042.8** | 0.72 |
| 5 | 42845.9 | 43268.0 | 43064.6 | 0.75 |
| 6 | **42826.1** | 43334.0 | 43089.3 | 0.73 |
|  |  |  |  |  |
| **Extension model** |  |  |  |  |
| **Age 13 classes** |  |  |  |  |
| 1 | 53001.0 | 53099.9 | 53052.2 | N/A |
| 2 | 50775.5 | 50980.0 | 50881.5 | 0.72 |
| 3 | 50035.0 | 50345.0 | 50195.6 | 0.81 |
| 4 | 49836.0 | **50251.5** | 50051.3 | 0.73 |
| 5 | 49770.0 | 50291.0 | 50040.0 | 0.73 |
| 6 | **49714.9** | 50341.5 | **50039.6** | 0.73 |
|  |  |  |  |  |
| **Comparable model** |  |  |  |  |
| **Age 17 classes** |  |  |  |  |
| 1 | 62259.7 | 62383.5 | 62323.1 | N/A |
| 2 | 59732.0 | 59986.1 | 59986.1 | 0.63 |
| 3 | 58731.3 | 59115.7 | 58928.2 | 0.74 |
| 4 | 58498.4 | **59013.1** | 58762.1 | 0.74 |
| 5 | 58413.6 | 59058.7 | 58744.1 | 0.71 |
| 6 | **58323.2** | 59098.5 | **58720.4** | 0.68 |
|  |  |  |  |  |
| **Extension model** |  |  |  |  |
| **Age 17 classes** |  |  |  |  |
| 1 | 70462.6 | 70599.5 | 70532.7 | N/A |
| 2 | 67717.6 | 67997.7 | 67861.1 | 0.64 |
| 3 | 66725.7 | 67149.2 | 66942.6 | 0.74 |
| 4 | 66462.8 | **67029.7** | 66753.2 | 0.74 |
| 5 | 66326.3 | 67036.5 | 66690.1 | 0.70 |
| 6 | **66246.2** | 67099.6 | **66683.5** | 0.70 |
|  |  |  |  |  |

**4. Latent class probabilities**

| **Table S5: Probability distributions for GUI latent classes at ages 13 and 17** | | | | | |
| --- | --- | --- | --- | --- | --- |
| **Probability distribution for age 13** | | | | | |
| **Name of latent class (approximate proportion of the sample)** | | **Family conflict and externalising problems (3.9%)** | **School and substance use problems (4.5%)** | **Peer problems (10.0%)** | **Low risk (81.7%)** |
| **Estimated female percentage** | | 34.4% | 38.5% | 54.3% | 49.7% |
| **Risk Factor** | |  |  |  |  |
| SDQ (internalising problems) | | 0.49 | 0.00 | 0.51 | 0.02 |
| Bullying victim | | 0.27 | 0.16 | 0.43 | 0.05 |
| Young person has chronic mental health problem | | 0.29 | 0.00 | 0.09 | 0.01 |
| Substance use | | 0.20 | 0.60 | 0.07 | 0.03 |
| Conflict with parents (Pianta score) | | 0.81 | 0.30 | 0.37 | 0.06 |
| Parental divorce or separation | | 0.14 | 0.18 | 0.13 | 0.04 |
| Unpopular with peers (Piers Harris) | | 0.39 | 0.56 | 0.70 | 0.27 |
| Involved in violence | | 0.18 | 0.33 | 0.03 | 0.02 |
| SDQ (externalising problems) | | 1.00 | 0.08 | 0.07 | 0.01 |
| Not from a professional or managerial SES | | 0.77 | 0.56 | 0.65 | 0.47 |
| Parents use physical punishment on young person | | 0.29 | 0.36 | 0.21 | 0.09 |
| Parent has chronic mental health problem | | 0.05 | 0.05 | 0.12 | 0.03 |
| Truancy or excessive absence from school | | 0.20 | 0.56 | 0.13 | 0.04 |
| **Probability distribution for age 17** | | | | | |
| **Name of latent class (approximate proportion of the sample)** | **Depression (diagnosed) and psychiatric illness (5.9%)** | **Depression (undiagnosed), bullied and high substance use (6.9%)** | **Depression (undiagnosed), bullied and low substance use (20.8%)** | **Low Risk (46.5%)** | **Moderate smoking and high alcohol use (19.8%)** |
| **Estimated female percentage** | 60.6% | 52.0% | 69.5% | 47.4% | 26.8% |
| **Risk Factor** |  |  |  |  |  |
| Diagnosed with depression or anxiety | 0.85 | 0.36 | 0.10 | 0.02 | 0.04 |
| Depression (SMFQ) | 0.67 | 0.83 | 0.73 | 0.08 | 0.13 |
| Bullying victim | 0.42 | 0.78 | 0.69 | 0.21 | 0.31 |
| Psychiatric or mental health issue | 0.68 | 0.23 | 0.02 | 0.01 | 0.00 |
| Smokes (daily or occasionally) | 0.23 | 0.83 | 0.14 | 0.01 | 0.50 |
| Uses alcohol | 0.38 | 0.78 | 0.36 | 0.24 | 0.77 |
| Uses cannabis or drugs | 0.18 | 0.76 | 0.05 | 0.03 | 0.35 |
| Poor family relationships | 0.07 | 0.19 | 0.08 | 0.05 | 0.07 |
| Parental divorce/separation | 0.10 | 0.32 | 0.12 | 0.06 | 0.10 |
| No close friend | 0.05 | 0.04 | 0.06 | 0.01 | 0.01 |
| Involved in violence | 0.08 | 0.43 | 0.12 | 0.05 | 0.31 |
| Externalising problems (SDQ) | 0.16 | 0.19 | 0.02 | 0.01 | 0.04 |
| Lower academic grades | 0.17 | 0.28 | 0.07 | 0.04 | 0.12 |
| Not from a professional or managerial SES | 0.59 | 0.53 | 0.51 | 0.49 | 0.58 |
| Experienced a break-up since age 13 | 0.39 | 0.69 | 0.45 | 0.23 | 0.53 |
| Parent has a chronic mental health illness | 0.10 | 0.04 | 0.03 | 0.02 | 0.03 |
| Has eating disorder (ESP) | 0.31 | 0.36 | 0.34 | 0.05 | 0.06 |
| Dropped out of school (without leaving certificate) | 0.06 | 0.01 | 0.00 | 0.00 | 0.00 |
| Truancy | 0.10 | 0.33 | 0.06 | 0.01 | 0.12 |
| LGBQA | 0.34 | 0.267 | 0.149 | 0.04 | 0.06 |

| **Probability distribution for extension model** | | | | |
| --- | --- | --- | --- | --- |
| **Name of latent class (approximate proportion of the sample)** | **Low risk (73.5%)** | **Peer problems (6.4%)** | **Substance use (15.5%)** | **Family conflict and psychiatric issues (4.6%)** |
| **Estimated female percentage** | 57.0% | 45.1% | 31.8% | 36.8% |
| **Risk Factor** |  |  |  |  |
| SDQ (internalising problems) | 0.02 | 0.5 | 0.03 | 0.31 |
| Bullying victim (between ages 5 and 11) | 0.14 | 0.57 | 0.3 | 0.4 |
| Psychiatric issue | 0 | 0.05 | 0.02 | 0.85 |
| Substance use | 0.18 | 0.15 | 0.71 | 0.53 |
| Poor family relations | 0.04 | 0.22 | 0.05 | 0.86 |
| Parental divorce or separation | 0.09 | 0.17 | 0.17 | 0.22 |
| Peer problems | 0.13 | 0.52 | 0.16 | 0.32 |
| Involved in violence | 0.07 | 0.23 | 0.56 | 0.39 |
| SDQ (externalising problems) | 0.01 | 0.15 | 0.05 | 0.46 |
| Not from a professional or managerial SES | 0.57 | 0.69 | 0.66 | 0.54 |
| Family history of psychiatric illness | 0.31 | 0.58 | 0.42 | 0.59 |
| Truancy | 0.01 | 0.06 | 0.34 | 0.21 |
| **Probability distribution for extension model** | | | | |
| **Name of latent class (approximate proportion of the sample)** | **Low risk (70.0%)** | **Peer problems (12.0%)** | **Substance use (13.9%)** | **Family conflict and psychiatric issues (4.0%)** |
| **Estimated female percentage** | 54.8% | 42.2% | 46.1% | 38.3% |
| **Risk Factor** |  |  |  |  |
| SDQ (internalising problems) | 0.02 | 0.3 | 0.03 | 0.33 |
| Bullying victim (between 5 and 11 years) | 0.14 | 0.48 | 0.25 | 0.42 |
| Psychiatric issue | 0 | 0.05 | 0.02 | 0.91 |
| Smoking | 0.02 | 0.03 | 0.44 | 0.21 |
| Use of alcohol (without parents’ permission) | 0.14 | 0.1 | 0.86 | 0.47 |
| Cannabis use | 0 | 0 | 0.25 | 0.15 |
| Poor family relations | 0.03 | 0.18 | 0.07 | 0.91 |
| Parental divorce | 0.09 | 0.18 | 0.16 | 0.23 |
| Peer problems | 0.12 | 0.42 | 0.15 | 0.34 |
| Involved in violence | 0.09 | 0.26 | 0.45 | 0.39 |
| SDQ (externalising problems) | 0.01 | 0.12 | 0.05 | 0.48 |
| Lower SES | 0.58 | 0.68 | 0.6 | 0.54 |
| Family history of psychiatric illness | 0.3 | 0.54 | 0.43 | 0.6 |
| Truancy | 0.02 | 0.09 | 0.3 | 0.21 |
| Childhood abuse | 0.07 | 0.23 | 0.11 | 0.25 |

| **Table S7: Probability distributions for age 17 ALSPAC latent classes at ages** | | | | |
| --- | --- | --- | --- | --- |
| **Probability distribution for extension model** | | | | |
| **Name of latent class (approximate proportion of the sample)** | **Depression and low substance use (10.3%)** | **Depression and high substance use (4.4%)** | **Low risk (63.7%)** | **Low depression and high substance use (21.6%)** |
| **Estimated female percentage** | 71.6% | 74.5% | 56.3% | 48.4% |
| **Risk Factor** |  |  |  |  |
| ICD-10 Diagnosis or symptoms of depression | 0.68 | 0.89 | 0.01 | 0.01 |
| Depression or anxiety (CIS) | 0.79 | 0.85 | 0.03 | 0.04 |
| History of bullying | 0.39 | 0.39 | 0.27 | 0.37 |
| Psychiatric illness | 0.13 | 0.23 | 0.03 | 0.12 |
| Smokes | 0.09 | 0.84 | 0.03 | 0.72 |
| Drinks | 0.34 | 0.82 | 0.26 | 0.75 |
| Cannabis | 0.01 | 0.45 | 0 | 0.3 |
| Drugs | 0.07 | 0.75 | 0.02 | 0.46 |
| Poor family relationships | 0.19 | 0.32 | 0.04 | 0.16 |
| Divorce | 0.03 | 0.05 | 0.03 | 0.06 |
| Unhappy with number of friends | 0.22 | 0.19 | 0.05 | 0.07 |
| Violence | 0.04 | 0.17 | 0.02 | 0.16 |
| SDQ (externalising problems) | 0.03 | 0.07 | 0.01 | 0.08 |
| Failed exams in schools | 0.23 | 0.23 | 0.13 | 0.18 |
| Not from a professional or managerial SES | 0.57 | 0.59 | 0.57 | 0.59 |
| Relationship break-up | 0.37 | 0.55 | 0.26 | 0.44 |
| History of psychiatric illness in family | 0.45 | 0.42 | 0.32 | 0.4 |
| Absenteeism or drop-out from school | 0.05 | 0.19 | 0.04 | 0.11 |
| LGBQA | 0.22 | 0.34 | 0.09 | 0.18 |
| **Probability distribution for extension model** | | | | |
| **Name of latent class (approximate proportion of the sample)** | **Depression and low substance use (10.9%)** | **Depression and high substance use (4.6%)** | **Low risk (62.9%)** | **Low depression and high substance use (21.7%)** |
| **Estimated female percentage** | 71.1% | 74.6% | 50.7% | 55.3% |
| **Risk Factor** |  |  |  |  |
| ICD-10 Diagnosis or symptoms of depression | 0.64 | 0.86 | 0 | 0.01 |
| Depression or anxiety (CIS) | 0.78 | 0.83 | 0.03 | 0.03 |
| History of bullying | 0.39 | 0.4 | 0.37 | 0.25 |
| Psychiatric illness | 0.13 | 0.24 | 0.12 | 0.02 |
| Smokes | 0.09 | 0.85 | 0.72 | 0.03 |
| Drinks | 0.33 | 0.82 | 0.74 | 0.26 |
| Cannabis | 0.01 | 0.44 | 0.29 | 0 |
| Drugs | 0.07 | 0.74 | 0.45 | 0.02 |
| Poor family relationships | 0.19 | 0.32 | 0.16 | 0.04 |
| Divorce | 0.03 | 0.05 | 0.06 | 0.03 |
| Unhappy with number of friends | 0.22 | 0.19 | 0.07 | 0.05 |
| Violence | 0.03 | 0.17 | 0.15 | 0.02 |
| SDQ (externalising problems) | 0.03 | 0.08 | 0.08 | 0.01 |
| Failed exams in schools | 0.23 | 0.23 | 0.17 | 0.13 |
| Not from a professional or managerial SES | 0.57 | 0.6 | 0.59 | 0.57 |
| Relationship break-up | 0.37 | 0.55 | 0.44 | 0.26 |
| History of psychiatric illness in family | 0.45 | 0.43 | 0.41 | 0.31 |
| Absenteeism or drop-out from school | 0.05 | 0.18 | 0.11 | 0.04 |
| LGBQA | 0.22 | 0.34 | 0.19 | 0.08 |
| Childhood abuse | 0.27 | 0.36 | 0.28 | 0.14 |
| Exposed to self-harm in family or friends | 0.62 | 0.85 | 0.55 | 0.35 |

**5. Bias assessment between those removed in TF2 and TF4**

Note that the Bonferroni correction was applied to the p-values for multiple testing

| S8: Comparing the proportions for those included and excluded in the TF2 and TF4 surveys | |
| --- | --- |
|  |  |
| **TF2 included (n=5,407) versus those removed (n=723)** | **p-value** |
| SDQ (internalising problems) | 0.1791 |
| Bullying victim (between ages 5 and 11) | 0.8775 |
| Psychiatric issue | 0.7617 |
| Substance use | **<0.001*** |
| Poor family relations | 0.0305 |
| Parental divorce or separation | 0.0257 |
| Peer problems | 0.0058 |
| Involved in violence | 0.6934 |
| SDQ (externalising problems) | 0.4655 |
| Not from a professional or managerial SES | 0.4635 |
| Family history of psychiatric illness | 0.0768 |
| Truancy | **0.0027*** |
|  |  |
| **TF4 included (n=5,210) versus those removed (n=195)** |  |
| ICD-10 Diagnosis or symptoms of depression | 0.5261 |
| Depression or anxiety (CIS) | 0.3082 |
| History of bullying | 0.466 |
| Psychiatric illness | 0.01723 |
| Smokes | 0.01486 |
| Drinks | 0.02151 |
| Cannabis | 0.03818 |
| Drugs | 0.01954 |
| Poor family relationships | 0.934 |
| Divorce | 0.2911 |
| Unhappy with number of friends | 0.292 |
| Violence | 0.1529 |
| SDQ (externalising problems) | **<0.001*** |
| Failed exams in schools | 0.1687 |
| Not from a professional or managerial SES | 0.7098 |
| Relationship break-up | 0.4312 |
| History of psychiatric illness in family | 0.3675 |
| Absenteeism or drop-out from school | 0.3799 |
| LGBQA | 0.7186 |
|  |  |
|  |  |
|  |  |

* Bonferroni correction at 0.05/12 = 0.004 level of significance

**6. References**

Angold, A., Costello, E. J., Messer, S. C., & Pickles, A., 1995. Development of a short questionnaire for use in epidemiological studies of depression in children and adolescents. International Journal of Methods in Psychiatric Research, 5(4), 237–249. <https://psycnet.apa.org/record/1996-02633-002>

Babor, T. F., Higgins-Biddle, J. C., Saunders, J. B. & Monteiro, M. G. 2001. AUDIT: The Alcohol Use Disorders Identification Test Guidelines for Use in Primary Care. Second Edition. Geneva, Switzerland. <https://www.who.int/publications/i/item/WHO-MSD-MSB-01.6a> [Accessed 16 June 2024]

Batejan, K.L., Jarvi, S.M., Swenson, L.P., 2015. Sexual orientation and non-suicidal self-injury: A meta-analytic review. Archives of Suicide Research 19(2), 131-150. <https://doi.org/10.1080/13811118.2014.957450>

Castellví, P., Miranda-Mendizábal, A., Alayo, I., Parés-Badell, O., Almenara, J., Alonso, I., Blasco, M.J., Cebrià, A., Gabilondo, A., Gili, M., Lagares, C., Piqueras, J.A., Roca, M., Rodríguez-Marín, J., Rodríguez-Jimenez, T., Soto-Sanz, V., Alonso, J., 2020. Assessing the relationship between school failure and suicidal behavior in adolescents and young adults: A systematic review and meta-analysis of longitudinal studies. School Mental Health: A Multidisciplinary Research and Practice Journal. 12(3), 429–441 <https://psycnet.apa.org/doi/10.1007/s12310-020-09363-0>

Cheek, S.M., Reiter-Lavery, T., Goldston, D.B., 2020. Social rejection, popularity, peer victimization, and self-injurious thoughts and behaviors among adolescents: A systematic review and meta-analysis. Clinical Psychology Review 82. 101936. <https://doi.org/10.1016/j.cpr.2020.101936>

Community-University Partnership For The Study Of Children Youth And Families 2011. Review of the Piers-Harris Children's Self-Concept Scale 2^nd^ Edition. Edmonton, Alberta, Canada. <https://www.ualberta.ca/community-university-partnership/media-library/community-university-partnership/resources/tools---assessment/piers-harris-2may-2012.pdf> [Accessed 1 April 2023]

Cotton, M. A., Ball, C. & Robinson, P. 2003. Four simple questions can help screen for eating disorders. Journal of General Internal Medicine, 18, 53-6. <https://doi.org/10.1046%2Fj.1525-1497.2003.20374.x>

Evans, R., White, J., Turley, R., Slater, T., Morgan, H., Strange, H., Scourfield, J., 2017. Comparison of suicidal ideation, suicide attempt and suicide in children and young people in care and non-care populations: Systematic review and meta-analysis of prevalence. Children and Youth Services Review 82, 122-129. <https://doi.org/10.1016/j.childyouth.2017.09.020>

Geulayov, G., Gunnell, D., Holmen, T.L., Metcalfe, C., 2012. The association of parental fatal and non-fatal suicidal behaviour with offspring suicidal behaviour and depression: A systematic review and meta-analysis. Psychological Medicine 42(8), 1567-1580. <https://doi.org/10.1017/S0033291711002753>

Goodman, A., Goodman, R., 2009. Strengths and difficulties questionnaire as a dimensional measure of child mental health. Journal of the American Academy of Child Adolescent Psychiatry, 48(4):400-403. <https://doi.org/10.1097/CHI.0b013e3181985068>

Goodman, A., Heiervang, E., Collishaw, S. & Goodman, R. 2011. The 'DAWBA bands' as an ordered-categorical measure of child mental health: description and validation in British and Norwegian samples. Social Psychiatry and Psychiatric Epidemiology, 46, 521-32 <https://doi.org/10.1007/s00127-010-0219-x>

Lewis, G., Pelosi, A. J., Araya, R. & Dunn, G. 1992. Measuring psychiatric disorder in the community: a standardized assessment for use by lay interviewers. Psychological Medicine, 22, 465-86. <https://doi.org/10.1017/s0033291700030415>

McEvoy, D., Brannigan, R., Cooke, L., Butler, E., Walsh, C., Arensman, E. & Clarke, M. 2023. Risk and protective factors for self-harm in adolescents and young adults: An umbrella review of systematic reviews. Journal of Psychiatric Research, 168, 353-380. <https://doi.org/10.1016/j.jpsychires.2023.10.017>

Miranda-Mendizábal, A., Castellví, P., Parés-Badell, O., Alayo, I., Almenara, J., Alonso, I., Blasco, M.J., Cebrià, A., Gabilondo, A., Gili, M., Lagares, C., Piqueras, J.A., Rodríguez-Jiménez, T., Rodríguez-Marín, J., Roca, M., Soto-Sanz, V., Vilagut, G., Alonso, J., 2019. Gender differences in suicidal behavior in adolescents and young adults: Systematic review and meta-analysis of longitudinal studies. International Journal of Public Health 64(2), 265-283. <https://doi.org/10.1007/s00038-018-1196-1>

Pianta, R. C. 2011. Pianta Child-Parent Relationship Scale [Online]. Available: <https://effectiveservices.force.com/s/measure/a007R00000v8QbbQAE/pianta-childparent-relationship-scale> [Accessed 17/04/2023].

Pozuelo, J.R., Desborough, L., Stein, A., Cipriani, A., 2021. Systematic review and meta-analysis: Depressive symptoms and risky behaviors among adolescents in Low- and Middle-income countries. Journal of the American Academy of Child & Adolescent Psychiatry 61(2):255-276. <https://doi.org/10.1016/j.jaac.2021.05.005>

Soto-Sanz, V., Piqueras, J.A., Rodriguez-Marin, J., Perez-Vazquez, M., Rodriguez-Jimenez, T., Castellvi, P., Miranda-Mendizábal, A., Pares-Badell, O., Almenara, J., Blanco, M.J., Cebria, A., Gabilondo, A., Gili, M., Roca, M., Lagares, C., Alonso, J., 2019. Self-esteem and suicidal behaviour in youth: A meta-analysis of longitudinal studies. Psicothema 31(3), 246-254. <https://doi.org/10.7334/psicothema2018.339>

World Health Organization, 2022. ICD-11 for Mortality and Morbidity Statistics (Version: 02/2022). <https://icd.who.int/browse11/l-m/en#!/http%3A%2F%2Fid.who.int%2Ficd%2Fentity%2F1354844071>. (Accessed 10/02/2023).

Zatti, C., Rosa, V., Barros, A., Valdivia, L., Calegaro, V.C., Freitas, L.H., Cereser, K.M.M., Rocha, N.S.d., Bastos, A.G., Schuch, F.B., 2017. Childhood trauma and suicide attempt: A meta-analysis of longitudinal studies from the last decade. Psychiatry Research 256, 353-358. <https://doi.org/10.1016/j.psychres.2017.06.082>
